# Prevalence, patterns, and disclosure of complementary and alternative medicine (CAM) use among patients with thyroid diseases: A cross-sectional study in Iran

**DOI:** 10.1016/j.heliyon.2024.e33436

**Published:** 2024-06-22

**Authors:** Mohadeseh Ostovar, Mesbah Shams, Marjan Mahmoudi, Azizallah Dehghan, Arezoo Moini Jazani, Mohammad Hashem Hashempur

**Affiliations:** a Research Center for Traditional Medicine and History of Medicine, Department of Persian Medicine, School of Medicine, Shiraz University of Medical Sciences, Shiraz, Iran; b Endocrinology and Metabolism Research Center, Shiraz University of Medical Sciences, Shiraz, Iran; c Student Research Committee, Shiraz University of Medical Sciences, Shiraz, Iran; d Noncommunicable Diseases Research Center, Fasa University of Medical Sciences, Fasa, Iran; e Traditional Medicine and Hydrotherapy Research Center, Ardabil University of Medical Sciences, Ardabil, Iran

**Keywords:** Complementary and alternative medicine, Hyperthyroidism, Hypothyroidism, Iran, Medicinal herbs, Thyroid diseases, Traditional Persian medicine

## Abstract

**Introduction:**

Complementary and alternative medicine (CAM) has gained popularity as a therapeutic approach outside conventional medicine for various medical conditions. This study aimed to assess the prevalence, patterns, and disclosure of CAM use among patients with thyroid diseases in Iran.

**Methods:**

This descriptive-analytic cross-sectional study involved patients with thyroid diseases who were visiting Internal Medicine Clinics in Shiraz. The use of CAM was assessed by employing the Persian edition of the I-CAM-Q (I-CAM-IR) questionnaire.

**Results:**

A total of 343 individuals took part in the study, and 85.4 % of them reported using CAM within the previous 12 months. Medicinal herbs were the most commonly used CAM modality (63 %). CAM use was primarily for enhancing overall well-being. Among self-care CAM practices, praying was the most frequently employed (70 %). Women exhibited a significantly higher CAM utilization rate (four times greater) compared to men (p < 0.001), with herbs being more commonly used by women (p < 0.001). A significant proportion of participants did not inform their physicians about their CAM use, and 46 % experienced significant benefits from using CAM.

**Conclusion:**

This study highlights a high prevalence of CAM use, particularly herbal remedies, among Iranian patients with thyroid diseases.

## 1 Introduction

Complementary and alternative medicine (CAM) encompasses therapeutic practices that fall outside the realm of conventional medicine and often draw from non-Western medical traditions [1]. The use of CAM has gained popularity worldwide and is increasingly being sought as a treatment option for various medical conditions [2,3]. Many research studies have investigated the prevalence of CAM utilization in various countries and populations [2,[4], [5], [6], [7], [8], [9]]. However, the data obtained from these studies exhibit significant variability, likely due to differences in methodology and the use of various questionnaires [10]. Iranians have a long-standing history of utilizing traditional medicine, making them familiar with the concept of CAM. Traditional Persian medicine (TPM), an ancient healthcare system that emphasizes lifestyle interventions, offers an effective approach to prevention, disease control, and treatment [11,12].

Thyroid diseases are a group of disorders that affect the thyroid gland. These diseases can range from benign conditions such as hypothyroidism and hyperthyroidism to more serious conditions like thyroid cancer. Given the chronic nature of thyroid diseases and the potential for long-term medication use, numerous patients seek CAM as a means of managing their symptoms and improving their overall well-being [[13], [14], [15], [16]].

CAM usage is assessed in both the general populace and individuals with particular health conditions across the globe. While some studies have explored CAM use in the general population of Iran, no research has focused specifically on CAM use among patients with thyroid diseases. A study conducted in Babol revealed that approximately 71 % of 1770 participants reported lifetime CAM use [17]. Similarly, a study in Kashan reported that 74 % of participants had a history of CAM use [18]. Another survey conducted in Khorramabad involving 790 subjects showed that approximately 80 % of individuals visiting health centers and hospitals had utilized at least one CAM method [19]. Additionally, a study involving 919 pregnant mothers in Mashhad found that approximately 84 % of them had used CAM during pregnancy [20].

Despite these findings, there is a scarcity of information on CAM usage specifically among patients with thyroid diseases, as existing studies have mainly concentrated on CAM use in individuals with thyroid cancer. For instance, Rosen et al. reported that about 74 % of thyroid cancer patients had utilized CAM [21]. However, data regarding CAM use among patients with other types of thyroid diseases remain scarce.

Therefore, the novelty of our work lies in the investigation of the use, patterns, and disclosure of CAM specifically among patients with thyroid diseases in Iran. To the best of our knowledge, there is limited research focusing on this specific population and region, making our study a unique contribution to the existing literature on CAM use in the context of thyroid diseases. Furthermore, our research question was investigated using the standardized Persian version of the I-CAM-Q questionnaire.

This research will contribute to our understanding of CAM utilization in this patient population and provide insights into the potential benefits and challenges associated with CAM therapies in managing thyroid diseases.

## 2 Methods

### 2.1 Study design

This cross-sectional descriptive-analytical study utilized the I-CAM-IR questionnaire (Supplementary file 1), which is the standard Persian version of the I-CAM-Q, an internationally recognized questionnaire on the use of CAM [1,22]. The I-CAM-Q is a self-administered questionnaire comprising four main sections designed to evaluate individuals' utilization of CAM.

The first section of the questionnaire assesses whether participants have used any form of CAM in the past 12 months (i.e. the 12-month period preceding each patient's completion of the questionnaire), their reasons for using it, the perceived helpfulness of CAM, and their disclosure to healthcare providers. The second section focuses on the modality of CAM received from different providers. The third section requires participants to specify the type of biological products they have used, such as herbs, homeopathic drugs, and vitamins, along with reasons for use and perceived helpfulness. The fourth and final section explores self-care CAM modalities employed by participants.

### 2.2 Setting

The study was conducted from January to March 2019 in three academic outpatient clinics (affiliated with Shiraz University of Medical Sciences) located in Shiraz, southern Iran.

#### 2.2.1 Participants

Patients diagnosed with thyroid diseases who voluntarily consented to participate in the study were enrolled. The diagnosis of thyroid diseases was made by an endocrinologist. Due to the absence of a comprehensive database specifically for these patients, we opted to utilize an interview-based approach to gather data from a convenient sample of attending patients. Patients with language or cognitive disorders, as well as those who incompletely filled out the questionnaires, were excluded.

### 2.3 Ethical considerations

The study received approval from the Local Medical Ethics Committee of Fasa University of Medical Sciences (ethics code: IR.FUMS.REC.1397.138) and adhered to the principles of the Declaration of Helsinki. Given the cross-sectional nature of the study and the self-administered questionnaire used, verbal informed consent was obtained from all participants, and measures were taken to ensure the confidentiality of their data.

#### 2.3.1 Sample size

The sample size was calculated using the formula: n = z^2^ × P × (1- P)/d^2^, with a confidence level of 95 % (z = 1.96), estimated prevalence of CAM use of 30 % (P = 0.3), and a margin of error of 0.05 (d = 0.05). Based on previous studies [23,24], the estimated sample size was 311. In order to increase the study power and account for the possibility of incomplete forms, a total of 343 patients were recruited.

### 2.4 Statistical analysis

Data obtained from the questionnaires were entered into the Statistical Package for Social Sciences (IBM Corp. Released 2019, IBM SPSS Statistics for Windows, Version 26.0, Armonk, NY) and analyzed using frequency analysis, *t*-tests, and cross-tabulation (chi-square tests).

## 3 Results

### 3.1 Demographic and clinical information

A total of 343 patients with thyroid diseases participated in the study. The demographic characteristics of the study cohort were presented in Table 1. Moreover, the most prevalent thyroid diseases among the participants were hypothyroidism (66.8 %), thyroid nodules (17.5 %), hyperthyroidism (12.8 %), and thyroid cancers (2.6 %). The most common co-morbid diseases reported by the participants were cardiovascular diseases (26.2 %), diabetes mellitus (12.5 %), renal diseases (6.4 %), and musculoskeletal disorders (6.1 %).

**Table 1:** The demographic characteristics of the study cohort

Mean Age, **years ± SD**		**43.93±14.03**
Gender, N (%)	Male	44 (12.8)
	Female	229 (87.2)
Place of birth, N (%)	Rural	20 (5.8)
	Urban	323 (94.2)
Education, N (%)	Illiterate	26 (7.6)
	High school	131 (38.2)
	Academic	186 (54.2)
Job, N (%)	Housewife	235 (68.5)
	Retired	24 (7)
	Unemployed	8 (2.3)
	Student	11 (3.2)
	employee	65 (19)
Sources of familiarity with CAM, N (%)^a^	Family and relatives	109 (31.8)
	Radio and television	107 (31.2)
	Digital platforms	92 (26.8)
	Training courses	24 (7)
	Books	51 (15.5)

SD: standard deviation; CAM: complementary and alternative medicine

a The combined sum of values may surpass 100% as patients were permitted to choose multiple options.

### 3.2 CAM use

The data obtained from the first section of the questionnaire, which focused on the CAM use in the last 12 months, including the types of CAM used, the main reasons for CAM use, the perceived helpfulness, and the disclosure of CAM use, are presented in Table 2. In the last 12 months, 293 participants (85.4 %), including 264 women and 29 men, reported using at least one form of CAM.

**Table 2:** Details of CAM use among patients with thyroid disorders (sorted in descending order).

Item	Usage, No (% of total participants^a^)	The main reason, No (%)			Helpfulness, No (%)				Disclosure, No (%)	
		For an acute disease	For a Chronic disease	To improve wellbeing	Very	Some what	Not at all	Don't know	Yes	No
**Herbs**	216 (63.0 %)	16 (7.5 %)	93 (43.0 %)	107 (49.5 %)	95 (44.0 %)	92 (42.6 %)	7(3.2 %)	22 (10.2 %)	200 (92.6 %)	16 (7.4 %)
**Vitamins**	193 (56.3 %)	4 (2.1 %)	104 (53.9 %)	85 (44.0 %)	65 (33.7 %)	64 (33.2 %)	6 (3.1 %)	58 (30.0 %)	170 (88 %)	24 (12.4 %)
**Spiritual therapy**	60 (17.5 %)	–	2 (3.3 %)	58 (96.7 %)	43 (71.7 %)	15 (25.0 %)	–	2 (3.3 %)	3 (5.0 %)	57 (95.0 %)
**Wet cupping and phlebotomy**	21 (6.1 %)	2 (9.5 %)	8 (38.1 %)	11 (52.4 %)	10 (47.6 %)	5 (23.8 %)	2 (9.5 %)	4 (19.0 %)	7 (33.3 %)	14 (66.7 %)
**Yoga**	18 (5.2 %)	7 (38.9 %)	1 (5.6 %)	10 (55.5 %)	15 (83.3 %)	2 (11.1 %)	1 (5.6 %)	–	2 (11.1 %)	16 (88.9 %)
**Traditional Persian medicine**	12 (3.5 %)	1 (8.3 %)	8 (66.7 %)	3 (25.0 %)	6 (50.0 %)	2 (16.7 %)	1 (8.3 %)	3 (25.0 %)	2 (16.7 %)	10 (83.3 %)
**Biotherapy**	10 (2.9 %)	1 (10.0 %)	8 (80.0 %)	1 (10.0 %)	8 (80.0 %)	1 (10.0 %)	–	1 (10.0 %)	4 (40.0 %)	6 (60.0 %)
**Cupping**	9 (2.6 %)	2 (22.2 %)	4 (44.5 %)	3 (33.3 %)	6 (66.7 %)	2	–	1	1	8
**Energy therapy**	5 (1.5 %)	–	1 (20.0 %)	4 (80.0 %)	4 (80.0 %)	1 (20.0 %)	–	–	–	5 (100 %)
**Acupuncture**	4 (1.2 %)	1 (25.0 %)	2 (50.0 %)	1 (25.0 %)	–	3 (75.0 %)	–	1 (25.0 %)	–	4 (100 %)
**Massage**	4 (1.2 %)	2 (50.0 %)	1 (25.0 %)	1 (25.0 %)	2 (50.0 %)	2 (50.0 %)	–	–	–	4 (100 %)
**Homeopathy**	0	–	–	–	–	–	–	–	–	–
**Tai-chi**	0	–	–	–	–	–	–	–	–	–

a The summation of the percentage values may be more than 100 % (the patients were allowed to choose more than one option).

**Fig. 1:**
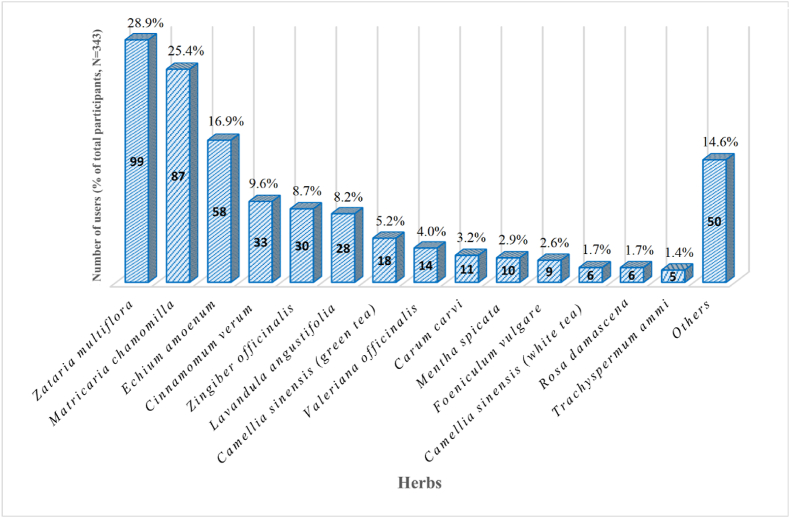
The most prevalent herbs used by the studied patients.

Herbal drug use was the most prevalent type of CAM reported by the participants. Among the participants who used herbal drugs, 107 used them to improve well-being, 93 for chronic diseases, and 16 for acute diseases. The helpfulness of using herbs was reported as "very" by 95 participants, "somewhat" by 92, and "not at all" by 7. Additionally, 22 participants reported that they were unsure of the helpfulness of using herbs. They mentioned using approximately 50 different herbs, with the most commonly used ones shown in Fig. 1.

**Fig. 2:**
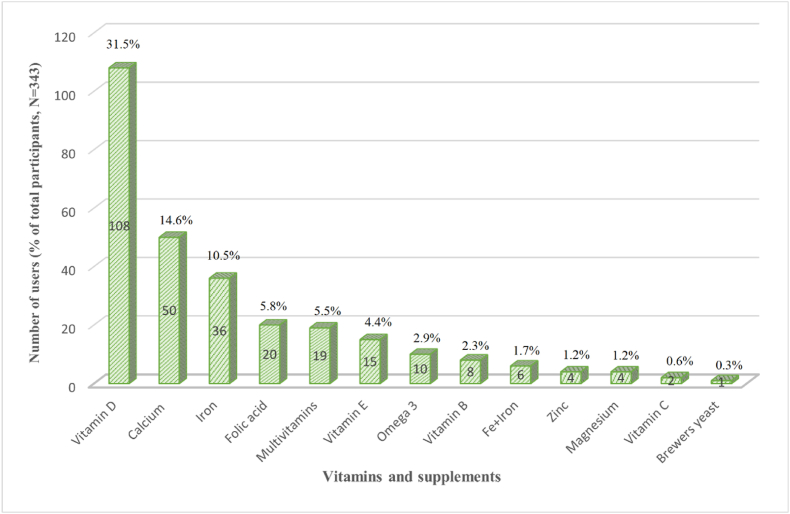
The most prevalent vitamins and supplements used by the studied patients.

The most commonly used vitamins and supplements are shown in Fig. 2. A total of 193 participants used vitamins mainly for chronic diseases (n = 104), 85 for well-being improvement, and 4 for acute diseases. The helpfulness of using vitamins was reported as "very" by 65 participants, "somewhat" by 64, and "not at all" by 6. Furthermore, 58 patients mentioned that they were unsure of the helpfulness of using vitamins.

Table 3 displays the types of CAM providers. None of the participants reported visiting a homeopath or chiropractor for CAM modalities. Table 4 presents data related to self-care CAM usage. Among self-care CAM practices, praying was the most frequently employed (70 %).

**Table 3:** The number of patients with thyroid diseases who received CAM modalities from each CAM provider.

Item	General Practitioner	Persian medicine Specialist	Herbalist (*Attar*)	Acupuncture Center	Chiropractor	Homeopath	Sport coach	Energy therapist	Religious Person	Others
Traditional Persian medicine	1	10	–	–	–	–	–	–	–	1
Homeopathy	–	–	–	–	–	–	–	–	–	–
Acupuncture	–	1	–	3	–	–	–	–	–	–
Wet cupping and phlebotomy	3	15	1	1	–	–	–	–	–	1
Cupping	–	6	–	–	–	–	–	–	–	3
Massage	–	–	–	–	–	–	1	–	–	3
Biotherapy	1	7	–	–	–	–	–	–	–	2
Herbs	7	9	136	–	–	–	–	–	–	64
Vitamins and supplements	170	1	1	–	–	–	–	–	–	21
Yoga	1	–	–	–	–	–	13	–	–	4
Tai-chi	–	–	–	–	–	–	–	–	–	–
Energy therapy	–	–	–	–	–	–	–	3	1	1
Spiritual therapy	–	–	–	–	–	–	1	–	29	30

**Table 4:** Details of self-help practices used among patients with thyroid disorders.

Item	Usage, No (% of total participants^a^)	The main reason, No (%)				Helpfulness, No (%)				Disclosure, No (%)	
		For an acute disease	For a Chronic disease	To improve wellbeing	Other	Very	Some what	Not at all	Don't know	Yes	No
**Praying for own health**	241 (70.3 %)	7 (2.9 %)	45 (18.7 %)	187 (77.6 %)	2 (0.8 %)	138 (57.3 %)	77 (32.0 %)	3 (1.2 %)	23 (9.5 %)	5 (2.1 %)	236 (97.9 %)
**Going to a religious place**	36 (10.5 %)	1 (2.8 %)	6 (16.7 %)	27 (75.0 %)	2 (5.5 %)	26 (72.3 %)	7 (19.4 %)	1 (2.7 %)	2 (5.6 %)	–	36 (100 %)
**Visualization**	16 (6.6 %)	2 (12.5 %)	7 (43.8 %)	5 (31.2 %)	2 (12.5 %)	14 (87.5 %)	2 (12.5 %)	–	–	3 (18.8 %)	13 (81.2 %)
**Attending traditional healing ceremony**	1 (0.3 %)	–	–	–	1 (100 %)	1 (100 %)	–	–	–	–	1 (100 %)
**Meditation**	23 (6.7 %)	–	–	10 (43.5 %)	13 (56.5 %)	20 (87.0 %)	2 (8.7 %)	1 (4.3 %)	–	4 (17.4 %)	19 (82.6 %)
**Relaxation techniques**	21 (6.1 %)	–	1 (4.8 %)	13 (61.9 %)	7 (33.3 %)	18 (85.7 %)	2 (9.5 %)	1 (4.8 %)	–	4 (19.1 %)	17 (80.9 %)
**Energy therapy**	5 (1.5 %)	–	1 (20.0 %)	4 (80.0 %)		–	4 (80.0 %)	1 (20.0 %)	–	–	5 (100.0 %)
**Yoga**	17 (5 %)	1 (5.9 %)	–	9 (52.9 %)	7 (41.2 %)	14 (82.3 %)	2 (11.8 %)	1 (5.9 %)	–	3 (17.3 %)	14 (82.3 %)
**Tai-chi or Qigong**	–	–	–	–		–	–	–	–	–	–
**Other**	1 (0.3 %)	–	–	1 (100.0 %)		–	1 (100.0 %)	–	–	–	1 (100.0 %)

a The summation of the percentage values may be more than 100 % (the patients were allowed to choose more than one option).

### 3.3 Associated factors for CAM use

There was a significant difference in CAM use between men and women (p < 0.0001). The use of wet cupping and phlebotomy, herbal drugs, and vitamins differed significantly between men and women (p < 0.02, p < 0.02, and p < 0.0001, respectively). There were no significant differences in CAM use based on the participants' birthplaces, except for Persian medicine and spiritual therapy.

## 4 Discussion

This study aimed to investigate the use of CAM among patients with thyroid diseases using the Persian version of the I-CAM-Q questionnaire (i.e. I-CAM-IR). It is the first study to explore CAM use specifically in patients with thyroid diseases. The study included 343 participants, and a high prevalence of CAM use was observed, with 85.4 % of participants reporting the use of at least one CAM modality in the past 12 months.

The prevalent tendency among Iranians to resort to alternative medicine reflects a broader cultural inclination towards holistic and natural approaches to health and healing. In Iran, traditional healing practices, have been deeply ingrained in the country's history and are often passed down through generations. Additionally, the accessibility and affordability of alternative medicine in Iran, further contribute to the widespread adoption of alternative therapies among the Iranian population [16,18,[25], [26], [27]].

When comparing the results of this study with prior research conducted in other nations, it becomes apparent that the utilization of CAM among patients with thyroid diseases differs. A study conducted in Boston on patients with thyroid cancer reported a CAM use prevalence of approximately 74 % (excluding multivitamin and prayer use) [28]. Another study on patients with thyroid nodules found that 79 % of participants had a history of CAM use [24].

In Iran, previous studies have shown a high prevalence of CAM use in the general population, ranging from 70 % to 85 % [[17], [18], [19],^29^]. The prevalence of CAM use also varied among specific disease populations, such as patients with diabetes mellitus (ranging from 37 % to 86 %), multiple sclerosis (67.9 %–85 %), dyslipidemia (77.4 %), thalassemia (68 %), coagulation disorders (49.3 %), cancer (35 %), and dermatological diseases (31 %). These differences in CAM use may be attributed to cultural factors, availability of CAM modalities, and the health status of individuals [23,[30], [31], [32], [33], [34], [35], [36], [37]]. Anbari and Ghanadi [38] reported a lifetime CAM use of approximately 80 % among clients over 15 years old referred to health centers and hospitals in *Khorramabad*. In the last year, the CAM use rate was found to be 58 % in the same population.

Similar studies conducted in other countries have reported varying rates of CAM use among the general population and patients. In a study conducted in the United States, CAM use was reported at 62 %, while an Australian research reported a rate of 48 %. Some other studies in the United Kingdom, Lao PDR, and England reported rates of 41 %, 40 %, and 26 % respectively, in the last year [[39], [40], [41], [42], [43]].

Various elements contribute to the differences in reported rates of CAM utilization among diverse populations. Firstly, cultural differences and the availability of various types of CAM play a significant role in determining CAM usage patterns. Secondly, healthy individuals tend to use CAM less frequently compared to patients. For example, a study conducted in Taiwan demonstrated that the rate of CAM use among participants increased from 23 % to 61 % after being diagnosed with diabetes mellitus [44]. Thirdly, factors such as the chronicity and prognosis of a disease can also influence the utilization of CAM. It is important to note that the comparability of data from these studies is limited due to variations in study methodologies and the use of different questionnaires to collect data on CAM use.

In this study, the most prevalent type of CAM used by participants was herbal drugs, which were primarily used to improve well-being. Herbal medicine has emerged as the most commonly embraced form of CAM among participants in Iran, reflecting a deep-rooted cultural significance and traditional healing practices in the country [17,18,30]. In Iranian culture, herbal medicine has been utilized for centuries as a natural and holistic approach to health and wellness. The rich history of herbal medicine in Iran, influenced by ancient Persian and Islamic traditions, has fostered a strong belief in the efficacy and safety of herbal remedies among the Iranian population. Moreover, the availability and accessibility of a wide variety of medicinal herbs in Iran's diverse climate and geography have further contributed to the widespread use of herbal medicine as a preferred CAM modality [[45], [46], [47], [48], [49], [50], [51]]. The prevalence of lifetime medicinal herb use in various studies conducted in Iran was reported as follows: 69.2 % in *Khorramabad* (western Iran), 68 % in *Kashan* (center), and 65.7 % in *Babol* (northern Iran).

*Zataria multiflora* and *Matricaria chamomila* were the most commonly used herbs for thyroid disorders in this study. Limited studies have reported the efficacy of these herbs on thyroid diseases, and further research is needed to confirm their effectiveness [52,53]. Other commonly used herbs, such as cinnamon and ginger, have shown potential efficacy in treating thyroid diseases, but additional studies are required for confirmation [[54], [55], [56]].

Self-care CAM use, including prayer, was reported by approximately 73 % of participants. Prayer and spiritual healing have been consistently reported as the most commonly used CAM modalities in various studies [57,58]. Iran is predominantly a Muslim-majority nation, with Shia Islam being the official state religion. The deeply rooted religious and spiritual beliefs of the Iranian population play a significant role in shaping their healthcare-seeking behaviors, including the high utilization of prayer and spiritual healing as forms of CAM. This high utilization rate may indeed be correlated with the minimal or nonexistent presence of an atheist population in Iran [[59], [60], [61], [62]].

The study had a higher participation rate of women compared to men, possibly reflecting the higher prevalence of thyroid diseases among females [63,64]. The women who participated in this study reported using CAM four times more than men, which is in line with previous research carried out in Iran. This may be attributed to cultural factors and the role of women in family healthcare [33,65,66].

The disclosure rate of CAM use to physicians was not low, with the highest disclosure rate observed for herbal drug use (92.6 %) followed by vitamins (87.6 %). Notably, a significant majority of patients (94.7 %) across all types of self-care CAM use refused to disclose their CAM usage to their physician. Furthermore, the complete lack of disclosure (rate of "0″) for acupuncture, massage, and energy therapy suggests that patients may be hesitant to discuss these modalities with their physicians. This finding highlights the need for improved communication between patients and healthcare professionals to prevent potential interactions between CAM and conventional treatments. Understanding the reasons behind this unwillingness to disclose CAM use is crucial in order to address any potential treatment interactions caused by physician unawareness.

Participants reported that the majority of CAM use was helpful, with approximately 46.0 % reporting it as "very helpful" (for self-care CAM 64.0 % of patients rated them "very helpful"). However, it should be noted that the helpfulness reported in this study was based on self-reporting by patients and does not confirm the efficacy of CAM modalities. Acupuncture was the least reported as "very helpful" with 75 % of participants reporting it as "somewhat helpful." Similarly, energy therapy was mentioned as "somewhat helpful" by 80 % of patients. These findings suggest that while patients perceive some benefit from these modalities, further research is needed to evaluate their efficacy. CAM use helpfulness was reported 70 % in a study which was carried out among diabetic patients in Palestine [67].

Despite the relatively narrow age range among participants in this study (43.93 ± 14.03), exploring the impact of age on CAM outcomes is a crucial aspect that warrants further investigation. Existing studies have indicated that age can influence individuals' preferences, beliefs, and behaviors regarding healthcare practices, including the utilization of CAM. Younger individuals may be more open to exploring alternative therapies, while older individuals may have different perspectives on traditional and complementary treatments [[68], [69], [70], [71]].

The insights gained from our research shed light on the utilization, patterns, and disclosure of CAM among patients with thyroid diseases, offering valuable information for healthcare providers and policymakers. The identification of prevalent herbal medicine use, motivations behind CAM utilization, and reported helpfulness of herbal remedies provide a nuanced understanding of patient preferences and behaviors. By incorporating these key findings into clinical practice, healthcare professionals can tailor treatment approaches to better meet the needs and preferences of patients with thyroid diseases. Moreover, the disclosure patterns revealed in our study can guide healthcare providers in fostering open communication with patients regarding their CAM usage, ultimately enhancing patient-centered care and promoting holistic well-being.

The present study has several strengths. We used an international questionnaire, which offered valuable insights into the utilization of CAM among individuals with thyroid diseases. Additionally, it is the first study to specifically investigate CAM use in this population. However, there are limitations to consider. The narrow age range of participants may limit the generalizability of the findings to a broader population. While our study provides valuable insights into the utilization patterns and motivations of older adults, the lack of younger individuals may overlook unique perspectives and practices related to CAM use in this demographic. Moreover, recall bias is a common problem in retrospective studies. The study also did not assess the harmful or beneficial effects of CAM use. Furthermore, the self-reported helpfulness of CAM use may not necessarily reflect its efficacy. Future studies should aim to evaluate the efficacy of CAM use in thyroid diseases. In addition, it is important to address the limitation regarding the ambiguity in participants' motives for using CAM, particularly in relation to managing thyroid disease versus other chronic health conditions. The study questionnaire (i.e., I-CAM-Q) did not explicitly differentiate between using CAM for thyroid disease management specifically or for other chronic conditions.

## 5 Conclusion

In conclusion, this study demonstrated a high prevalence of CAM use, especially herbs, among Iranian patients with thyroid diseases. CAM use was more popular among women than men, and a significant number of patients reported that CAM usage was helpful. Patients primarily used CAM to improve their well-being, often motivated by recommendations from friends and family members rather than healthcare professionals. The relatively low disclosure rate of CAM use to physicians highlights the need for improved communication in order to prevent potential treatment interactions. Further research is needed to evaluate the efficacy of CAM use in thyroid diseases and address the limitations of this study.

## Ethics statement

The study received approval from the Local Medical Ethics Committee of Fasa University of Medical Sciences (ethics code: IR.FUMS.REC.1397.138). Given the cross-sectional nature of the study and the self-administered questionnaire used, verbal informed consent was obtained from all participants.

## Funding

This study was financially supported by a grant from 10.13039/501100006402Fasa University of Medical Sciences (grant No. 97229).

## Data availability statement

Data will be made available on reasonable request from the corresponding author.

## CRediT authorship contribution statement

**Mohadeseh Ostovar:** Writing – review & editing, Writing – original draft, Supervision. **Mesbah Shams:** Writing – review & editing, Supervision, Resources, Methodology. **Marjan Mahmoudi:** Writing – review & editing, Investigation, Formal analysis, Data curation. **Azizallah Dehghan:** Writing – review & editing, Validation, Software, Methodology, Formal analysis, Data curation. **Arezoo Moini Jazani:** Writing – review & editing, Visualization, Supervision, Investigation. **Mohammad Hashem Hashempur:** Writing – review & editing, Validation, Project administration, Investigation, Data curation, Conceptualization.

## Declaration of competing interest

The authors declare that they have no known competing financial interests or personal relationships that could have appeared to influence the work reported in this paper.
