# Supplementary material for: Prevalence, patterns, and disclosure of complementary and alternative medicine (CAM) use among patients with thyroid diseases: A cross-sectional study in Iran
Source: Heliyon. 2024 Jun 22;10(13):e33436. doi: 10.1016/j.heliyon.2024.e33436 (PMC11260944; doi:10.1016/j.heliyon.2024.e33436)
Supplement: Multimedia component 1 [file mmc1.pdf]

1. Complementary and alternative Medicine (CAM) received in the past 12 months.

Have you used any of the following practices in the last 12 months?	Yes	No	Number of times you used this practice in the last 3 months?	Please indicate the main reason that applies to your last use (Check only one).				How helpful did you find this product? (Check only one)				Did you disclose to your physician?	
				For an acute illness/condition, one that lasted less than one month	To treat a long-term health condition (one that lasted more than one month) or its symptoms	To improve well-being	Other (Please specify)	Very	Somewhat	Not at all	Do not know	Yes	No
Traditional Persian Medicine													
Manipulation													
Homeopathy													
Acupuncture													
Herbs/Herbal therapy													
Dry-Cupping													
Wet-cupping/ Phlebotomy …													
Chiropractic													
Biotherapy (Leech/Maggot/Bee, …)													
Vitamins/supplements													
Yoga													
Tai chi													
Spiritual healing													
Energy therapy													
Other (please specify):													

2. Visiting health care provider/s: If you have not seen any of the health care providers in the past 12 months, please go to section 3.

It should be noted that CAM may be addressed by different groups of health care providers.

Please specify the health care provider/s from whom you received any of the following services:	General practitioner or other physicians	Persian medicine Specialist	Attar or herbalist	Acupuncturist	Chiropractor	Homeopath	Yoga trainer	Energy therapist	Religious Person	Others (by self or relatives)
Traditional Persian Medicine										
Manipulation										
Homeopathy										
Acupuncture										
Herbs/Herbal therapy										
Dry-Cupping										
Wet-cupping/ Phlebotomy, …										
Chiropractic										
Biotherapy (Leech/Maggot/Bee, …)										
Vitamins/supplements										
Yoga										
Tai chi										
Spiritual healing										
Energy therapy										
Other (please specify):										

3. Use of Herbal Medicine and Dietary Supplements, including tablets, capsules and liquids.

For each category below, please list up to three products you have used in the last 12 months.	Do you <u>currently</u> use this product?		Please indicate the <u>main</u> reason that applies to your <u>last</u> use (Check only one).				How helpful did you find this product? (Check only one)				Did you disclose to your physician?	
	Yes	No	For an acute illness/condition, one that lasted less than one month	To treat a long-term health condition (one that lasted more than one month) or its symptoms	To improve well-being	Other (Please specify)	Very	Somewhat	Not at all	Do not know	Yes	No
**Herbs/Herbal medicine**												
**Vitamins/Minerals**												
**Homeopathic remedies**												
**Other supplements**												

4. Self Help Practices

That is, any activity that is done arbitrarily to improve the level of health or disease without consulting a doctor.

Have you used any of the following self-help practices in the last 12 months?	Yes	No	Number of times you used this practice in the last 3 months?	Please indicate the main reason that applies to your last use (Check only one).				How helpful did you find this product? (Check only one)				Did you disclose to your physician?	
				For an acute illness/condition, one that lasted less than one month	To treat a long-term health condition (one that lasted more than one month) or its symptoms	To improve well-being	Other (Please specify)	Very	Somewhat	Not at all	Do not know	Yes	No
Meditation													
Yoga													
Tai chi													
Relaxation techniques													
Visualization													
Attending traditional healing ceremony													
Praying for own health													
Going to a religious place													
Energy therapy													
Others (please specify)													
